# Central Nervous System Histoplasmosis: An Updated Insight

**DOI:** 10.3390/pathogens12050681

**Published:** 2023-05-05

**Authors:** José Antonio Ramírez, María del Rocío Reyes-Montes, Gabriela Rodríguez-Arellanes, Armando Pérez-Torres, Maria Lucia Taylor

**Affiliations:** 1 Unidad de Micología, Departamento de Microbiología-Parasitología, Facultad de Medicina, Universidad Nacional Autónoma de México (UNAM), CDMX, Mexico City 04510, Mexico; 2 Departamento de Biología Celular y Tisular, Facultad de Medicina, Universidad Nacional Autónoma de México (UNAM), CDMX, Mexico City 04510, Mexico

**Keywords:** *H. capsulatum*, dissemination, CNS infection, histoplasmomas, host’s CNS injuries

## Abstract

Histoplasmosis is one of the systemic mycoses that can involve the Central Nervous System (CNS), and it is caused by the dimorphic ascomycete species of the *Histoplasma capsulatum* complex. Once in the CNS, this pathogen causes life-threatening injuries that are associated with clinical manifestations of meningitis, focal lesions (abscesses, histoplasmomas), and spinal cord injuries. The present review provides updated data and highlights a particular vision regarding this mycosis and its causative agent, as well as its epidemiology, clinical forms, pathogenesis, diagnosis, and therapy, focusing on the CNS.

## 1. Introduction

Histoplasmosis is a respiratory–systemic mycosis, developing from an auto-limited and benign respiratory infection to a life-threatening disseminated disease form. The most severe symptomatic infections occur in the setting of immunosuppression. Acute infection in immunocompetent hosts is usually resolved; however, this infection can be associated with certain professional activities of persons related to mining, archaeology, guano collection, eco-tourism, and the remodeling and demolition of houses, in which they are exposed to aerosols containing the fungal infective propagules growing in bat or bird droppings [1].

The disease evolves to a chronic form that develops a tissue reaction represented by a well-organized cytoarchitecture known as granuloma. The infection is produced by dimorphic ascomycetes of the *Histoplasma capsulatum* complex.

The outcome of histoplasmosis depends on the coordinated action of innate and adaptive arms of the host immune system, the multiplicity of infective propagules of its etiologic agent, as well as the virulence and the phylogenetic species of the fungal strains. Overall, individuals develop an infected benign form; however, under some circumstances, the infection evolves with particular clinical manifestations of the histoplasmosis depending on the affected organ. This is the case for frequent involvement of the lungs, spleen, and liver, contrasting with infrequent reports for that of the Central Nervous System (CNS) in patients who present a progressive disseminated histoplasmosis.

The original purpose of this review is quite essential because it attempts to highlight a narrow relationship between *H. capsulatum* infection and the risk of developing CNS involvements.

## 2. *H. capsulatum*

The fungus *H. capsulatum* grows at 25–28 °C in nature in its saprobic (infective) multicellular mycelial phase (M-phase), under special environmental conditions. The M-phase microscopic morphological characteristics of this heterothallic ascomycete, such as thin hyphae, microconidia, and tuberculous macroconidia, allow for its recognition in its asexual (anamorph) state. The sexual state (teleomorph) of this fungus is short-lived and is the result of its mating in different natural or experimental circumstances, and it corresponds to *Ajellomyces capsulatus*, which generates haploid spores (ascospores) after a meiosis process [2].

Inhalations of the infective M-phase propagules are the cause of the establishment of the infection and disease progression. In the infected susceptible host, the pathogen develops a thermo-dimorphic transition to a unicellular yeast phase (Y-phase), which is the parasitic and also the virulent morphological phase of the fungus, mainly replicating intracellularly in host cells of the mononuclear phagocytic system.

*H. capsulatum* is considered a complex of cryptic species, consisting of various groups of isolates that differ genetically and correlate with a particular geographic distribution. It has been isolated from five continents, and currently, based on several genetic diversity studies, the phylogeography of the *H. capsulatum* complex has been reorganized to at least 14 phylogenetic species and four lone lineages, which are distributed worldwide. In the Americas, 11 phylogenetic species have been highlighted (NAm 1; NAm 2; NAm 3; LAm A1; LAm A2; LAm B1; LAm B2; LAm C; LAm D; LAm E; and RJ), which, together with Australian, Netherlands, and African phylogenetic species form, at the moment, the *H. capsulatum* complex [3,4,5,6,7]. New adjustments to the *H. capsulatum* taxonomy were published by Sepúlveda et al. [8], who used a concatenated phylogenetic reconstruction in a whole genome assembly study. They renamed four *Histoplasma* geographical clusters from the American continent, previously identified by Kasuga et al. [3], as: *H. capsulatum sensu stricto* Darling 1906 (instead of lineage H81 from Panama); *H. mississippiense* sp. nov. (instead of NAm 1); *H. ohiense* sp. nov. (instead of NAm 2); and *H. suramericanum* sp. nov. (instead of LAm A). However, Voorhies et al. [9] consider that there are some misinterpretations in these types of whole genome assembly studies due to genome fragmentation, which have led to underutilization of the genome-scale data. Thus, an accurate genetic approach was implemented by Voorhies et al. [9] to avoid these inconveniences, whereby they analyzed five well-known *Histoplasma* strains from different phylogenetic groups based on their chromosome-level genome assembly, and they found several repeat gene regions and largely conserved gene orders within the chromosomes, which is in contrast to previous genome assembly studies [9].

## 3. Histoplasmosis

### 3.1. Epidemiology of Histoplasmosis

The real incidence of histoplasmosis around the world depends on whether its notification is mandatory or not in different countries. Traditionally, the epidemiological data come from reports using the histoplasmin skin test (HST), which indicates immune contact with its causative agent. Today, molecular tools have been implemented to detect the fungal presence in samples from nature or as an aid in the diagnosis of histoplasmosis [10,11,12,13].

In the Americas, the prevalence of the disease varies among different regions in the continent, being highly recurrent in Mexico, where outbreaks of histoplasmosis have occurred more frequently. In Europe, *Histoplasma* infections detected by positive HST in human populations in Italy [14], as well as fungal detection in different tissues of badgers [15], of a hedgehog [16] in Germany, and of a bat captured in France [17], support the existence of favorable ecological niches for *H. capsulatum* in Europe, where this mycosis is considered an emerging disease due to an increase in immigration and European tourism towards the American and African continents [18,19,20,21]. In Asia, Africa, and Australia the occurrence of indigenous histoplasmosis has been widely recognized [22,23,24,25].

Regarding the frequency of CNS histoplasmosis in the world, clinical manifestations and mortality are very low in patients with disseminated histoplasmosis, which is in agreement with data reported in the United States by Wheat et al. [26]. Although CNS histoplasmosis is more prevalent in immunocompromised patients, about 20–30% of CNS involvements have been associated with patients without immune system disorders [26,27].

### 3.2. Clinical Forms

Because the most common portal of entry for *H. capsulatum* infective propagules is the upper respiratory tract, the lung is its main target organ, developing a localized clinical form known as primary pulmonary histoplasmosis.

**Figure 1:**
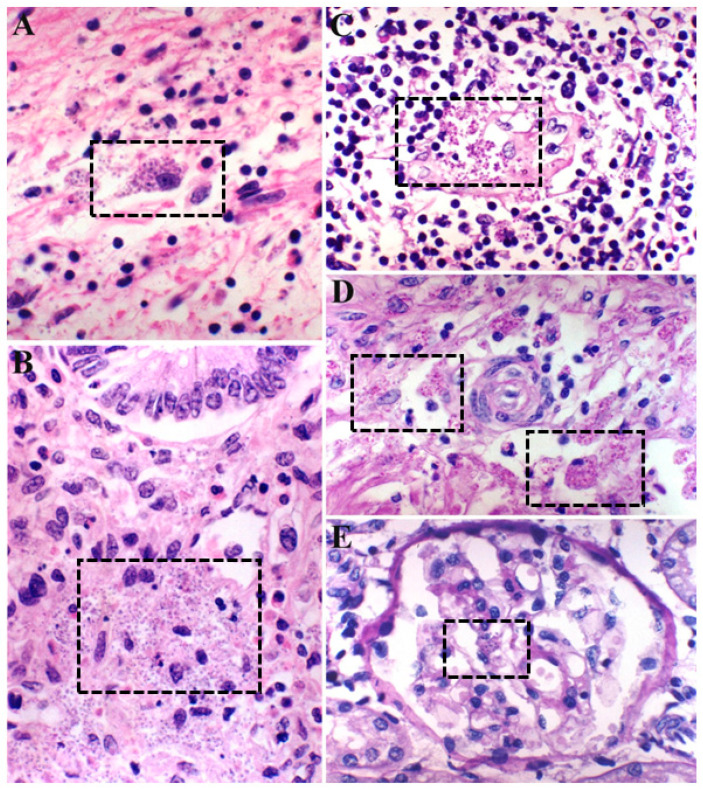
Disseminated histoplasmosis in an immunocompromised patient with AIDS. Note the presence of numerous intracellular and free yeast cells (dashed rectangles) in the ovary (**A**); lamina propria of the small intestine (**B**); paracortex of peripancreatic (**C**) and mesenteric lymph nodes (**D**); as well as in some mesangial cells of a renal glomerulus (**E**). Hematoxylin and eosin stain (**A**,**B**) and periodic acid-Schiff method (**C**–**E**). (**A**,**C**,**D**) 160×; (**B**,**E**) 200×.

Most infected individuals present an asymptomatic, self-limiting infection (benign form). In some cases, an acute infection can evolve with clinical manifestations of the disease that are generally resolved with the development of the host-cell-mediated immunity [28], leaving residual calcifications in the lungs and, sometimes, in the spleen (mild form). Although *H. capsulatum* is a primary pathogen, in patients with alterations of the immune response, mainly in cellular arms, *H. capsulatum* can act as an opportunist fungus, in which case the infection is usually not controlled, producing a progressive pulmonary clinical form that leads to severe disseminated histoplasmosis disease [29,30]. This severe clinical form of histoplasmosis can also affect immunocompetent individuals exposed to a high multiplicity of infective fungal propagules or to a highly virulent genotype of the *H. capsulatum* strains. Overall, this fungus affects the liver, kidney, and all secondary lymphoid organs (spleen, lymph nodes, and the mucosa-associated lymphoid tissues), reaching them through hematogenous and lymphatic routes (Figure 1).

Histoplasmosis affecting the CNS is less frequent than other infections produced by *H. capsulatum*, but data regarding these infections are generally available in case reports and medical literature reviews [31,32,33]. In particular, CNS histoplasmosis has been considered a consequence of fungal dissemination in infected individuals who have had a decrease in their immune response associated with immunosuppressive diseases or with procedures using immunosuppressive drugs [26,34]. In contrast, cases of CNS histoplasmosis are sporadic in immunocompetent hosts, and it has also been reported in no disseminated histoplasmosis [27,35,36]. The fungus can reach the CNS mainly by the hematogenous route and, rarely, by traumatism or inoculation during neurosurgical procedures [31,33].

**Figure 2:**
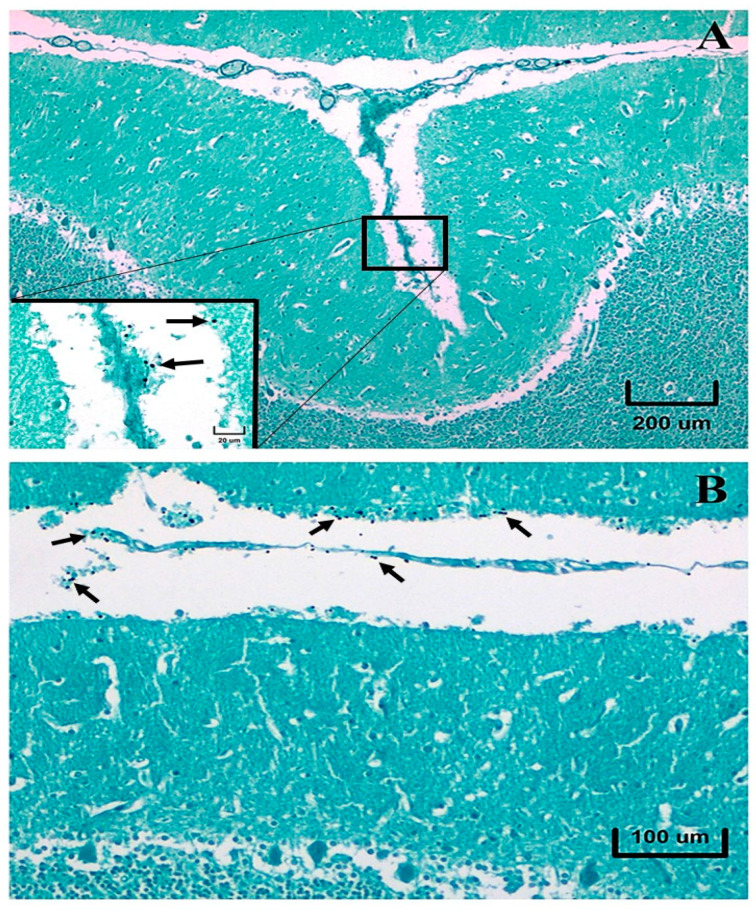
Human cerebellar cortex and sulci with leptomeninges (arachnoid and pia mater). Note the presence of yeast cells on the cortex surface and adhered to leptomeninges ((**A**), inset, arrows). Numerous yeast cells adhered to the same structures in other parts of the cerebellum ((**B**), arrows). Grocott methenamine silver stain.

The clinical forms of histoplasmosis in CNS are non-specific and depend on the affected site [37]. Symptoms in CNS histoplasmosis are subacute for several months, and only low percentages of the patients present symptoms in less than a month. The signs and symptoms presented by patients with CNS histoplasmosis include fever, headaches, lethargy, altered mental status, weakness, hydrocephalus, and focal neurological deficits that resemble cerebrovascular accidents [26,38]. CNS histoplasmosis patients can develop subacute or chronic lymphocytic meningitis (the most common), diffuse encephalitis, cerebral embolism, and spinal cord injuries [32,39,40,41]. In patients with cerebrospinal meningitis, the basilar meninges are the most severely affected area (Figure 2), and fluid pleocytosis can be observed [39,40,41,42].

In general, focal lesions associated with parenchymal masses in the brain or spinal cord (abscesses, histoplasmomas) in the course of CNS histoplasmosis are unusual [43,44]. The histoplasmomas in the CNS are frequently non-caseating granulomas, and they take the form of miliary granulomas [45,46]. In more advanced stages, the neurological status worsens with hindbrain signs, such as bilateral facial paralysis, dysarthria, and dysphagia, increasing the risk of aspiration [47].

### 3.3. Pathogenesis

In general, pathogenicity mechanisms of intracellular *Histoplasma* yeasts are mediated by virulence factors that act on a particular host target and can modulate or alter the patterns of the innate and adaptive immune responses of the infected hosts, promoting the participation of several host processes in the etiopathogenesis of the histoplasmosis.

Immune control of *Histoplasma* infections is administered by a complex network of events and signals from different subpopulations of CD4^+^ T lymphocytes (Th1, Th2, Th17, and Treg) as well as by CD8^+^ T lymphocytes, which with their respective cytokines stimulate the cellular immune response. In histoplasmosis, the protective immune response is mediated by Th1 lymphocytes, responsible for the production of pro-inflammatory cytokines, such as: IL-2, IFN-γ, IL-12, IL-18, TNF-α, CCL3, CCL5, CCL2, and CXCL8. Meanwhile, the non-protective immune response mediated by Th2 lymphocytes that produce the anti-inflammatory cytokines IL-4, IL-10, IL-5, IL-6, IL-13, and TGF-β can antagonize the pro-inflammatory response, occasionally fostering disease progression. However, in histoplasmosis, pro-inflammatory and anti-inflammatory responses need to be well-controlled in order to maintain an effective host defense. The absence of optimal pathogen clearance and its constant presence in macrophages can lead to non-controlled stimulation of the host immune response producing several injurious processes, which are the cause and effect in the pathology associated with intracellular diseases, such as histoplasmosis.

Although granuloma formation plays a protective role in the infected host by avoiding the progression of the fungal infection, sometimes, depending on the course of some chronic infections, the granuloma can subvert the host defense by involving several organs, leading to tissue damage with fibrotic processes. Furthermore, granulomas produced by *H. capsulatum* infection develop hypoxic microenvironments, which favor the infectious process [48].

There are few studies that define the pathogenesis of CNS histoplasmosis. The damaged areas in the CNS are located superficially in the meninges, in the brain tissue as focal lesions, or even as small cerebral infarcts [37,49]. Unusually, *Histoplasma*-associated post-infection inflammatory response syndrome was documented in the cerebrospinal fluid (CSF) through the determination of cytokines revealing elevated IL-6 and IL-8 levels [50].

### 3.4. Diagnosis

The diagnosis of patients with disseminated histoplasmosis is relatively accessible in specialized laboratories in countries where histoplasmosis is considered an epidemiological problem. *Histoplasma* dissemination can be identified in multiple organs; however, there are some difficulties in the diagnosis of patients with CNS involvement because samples are primarily obtained from the CSF, the meninges, or the nervous tissue and to achieve them it is necessary to employ invasive procedures.

Multiple approaches are recommended for the diagnosis of the different clinical forms of histoplasmosis, including laboratory tests, and histopathological, mycological, and serological evaluations. Radiologic images of CNS histoplasmosis have also been useful and vary from ring-enhancing cortical lesions to large masses or enhanced meningeal signals [37]. The demonstration of yeast cells (mainly intracellularly) on pathologic clinical samples together with fungal isolation in cultures, are undoubtedly indicative of histoplasmosis infection.

Diagnosis of CNS histoplasmosis is a great challenge for specialists. The CSF cultures are positive only in a few patients, and they show a delay in the results after the presentation of the clinical picture. For the isolation of the fungus in CNS histoplasmosis, it is recommended to employ appropriate volumes of CSF to facilitate the growth of *Histoplasma* colonies in a special mycological medium after long-term incubation at 28 and 37 °C [51]. Despite its low sensitivity, *H. capsulatum* isolation in cultures remains the gold standard for the diagnosis of histoplasmosis in the CSF or in the nervous tissue [31,36,40].

Faster diagnoses could be achieved by detecting antibodies and circulating fungal antigens in the CSF, which have shown greater positivity in patients with CNS histoplasmosis [52].

In particular, the serological tests for antibody detection present restrictions associated with false positive results due to the cross-reactivity of antibodies that recognize similar or shared antigens of other pathogens, which cause misinterpretations in the diagnosis as a result of the decreased specificity of the test used [26]. Moreover, an important limitation of serological tests is their use on samples from patients whose humoral immune response is diminished due to immunosuppressive conditions as a result of different causes.

In most cases of CNS histoplasmosis, the enzyme-linked immunosorbent assay (ELISA) is effectively used for antigen detection in urine, serum, CSF, and bronchoalveolar lavage samples [53,54]. In the clinical form of meningitis by *H. capsulatum*, higher levels of antigens have been shown in the CSF than in serum and urine samples, which suggests the presence of fungal antigens within the CNS due to a dissemination process through the blood–brain barrier [51].

Thus, CNS histoplasmosis must always be considered in the differential diagnosis of patients with subacute or chronic meningitis, and it is recommended to implement the ELISA test for the detection of circulating antigens and immunoglobulins (mainly G and M isotypes) in the CSF [52].

Urine *Histoplasma* antigen detection tests have revealed excellent performance for the rapid diagnosis of different histoplasmosis clinical forms, which could impact the diagnosis and treatment of patients. They show some advantages over molecular tests, such as being commercially available and easy to perform, and providing very fast results [54].

To date, several methods have been developed based on the polymerase chain reaction technique that employs diverse molecular markers for *H. capsulatum* detection in different clinical samples [54,55,56,57,58,59,60,61,62,63,64]; however, the use of molecular tests in CNS samples is scarce [63]. Molecular approaches offer the advantage of high analytical specificity and sensitivity, combined with these assays taking less time to provide results than those offered by other diagnostic methods [63]. Unfortunately, to date there has been no approval by the U.S. Food and Drug Administration regarding the use of molecular assays for *H. capsulatum* applied directly to clinical specimens [65].

### 3.5. Therapy

There are not enough comparative trials of CNS histoplasmosis therapy, and considering the scarcity and variability of clinical cases, it can occasionally be difficult to establish definitive treatments [66]. Amphotericin B, fluconazole, and itraconazole are used alone or in a combined form. Overall, the therapy must be aggressive and prolonged. Liposomal amphotericin B is preferred over standard amphotericin B formulation in view of its greater CNS penetration and lower toxicity. According to clinical practice, drug administration should be followed by clinical experience and descriptive studies [35,66]. To avoid relapses, clinical surveillance of patients should be monitored for at least 1 year after finishing treatment [26].

Other azoles, i.e., voriconazole, are also being considered in CNS histoplasmosis [67]). As long as there are no sufficient studies to establish a therapeutic scheme against CNS histoplasmosis, doses of azoles that are less aggressive for patients are a good alternative, mainly in immunocompromised individuals who require treatment for life [68].

## 4. Conclusions

CNS histoplasmosis is not a common clinical form of histoplasmosis, occurring only in 10% to 20% of disseminated histoplasmosis cases. However, it is associated with high mortality rates, even in the context of aggressive treatments. Given that CNS histoplasmosis has multiple clinical manifestations and no typical clinical picture, which is further combined by the diagnostic difficulties caused by the low sensitivity of laboratory tests, clinical suspicion of CNS histoplasmosis is low. This leads to erroneous diagnoses, resulting in chronic untreated infections with high morbidity and mortality. As a consequence, it is recommended to consider the presence of *H. capsulatum* in patients with unexplained neurological symptoms, chronic meningitis, or parenchymatous lesions of unknown etiology, particularly when other infectious causes such as tuberculosis and cryptococcosis have been ruled out.

Histoplasmosis is a significant health issue worldwide, and *Histoplasma* has become a genetic model system for experts in microorganisms that involve different hosts. Pandemic circumstances and the increasing use of immunosuppressive medications may have contributed to the spread of histoplasmosis globally, but many aspects regarding the natural history, environmental changes, and genetic structure of *Histoplasma* remain poorly understood, as well as the current status of different clinical manifestations of the disease. In this setting, CNS histoplasmosis could emerge as a new health challenge. From our point of view, our paper provides an update on this historically relegated manifestation in clinical practice.

## Data Availability

Not applicable.
